# Closed-Incision Negative Pressure Therapy in Place of Surgical Drain Placement in Plantar Fibroma Excision Surgery: A Case Series

**DOI:** 10.7759/cureus.9110

**Published:** 2020-07-10

**Authors:** Abbey Karlock, Ralph J Napolitano

**Affiliations:** 1 Osteopathic Medicine, Ohio University Heritage College of Osteopathic Medicine, Athens, USA; 2 Podiatry and Wound Care, OrthoNeuro, Columbus, USA

**Keywords:** plantar fibroma, foot tumors, podiatry, wound care, negative pressure wound therapy, prevena, closed-incision negative pressure wound therapy

## Abstract

Plantar fibromas are benign masses of fibrous tissue that develop in the arch of the foot arising from the plantar fascia. Symptomatology varies and is often related to weight bearing anatomic correlations or impingement of neurological or musculoskeletal structures. Several treatment options are available and include palliative measures, non-operative interventions and surgery, all with varying degrees of success and complication risk. The aim of this case study series was to assess surgical wound healing retrospectively in three patients who underwent wide en bloc plantar fibroma excision surgery for symptomatic lesions. Their incision was managed with a disposable closed-incision negative pressure therapy device (Prevena™; 3M + KCI, St. Paul, MN) in lieu of surgical drains. All three patients demonstrated favorable outcomes without complications.

## Introduction

Epidemiology and pathophysiology

Plantar fibromatosis (PF) is a rare disease characterized by nodules in the plantar fascia caused by disordered hyperproliferation of the fibrous tissue. It was first recorded by George Ledderhose in 1897 and is also referred to as Ledderhose disease [1].

The National Institutes of Health classify PF as a “rare disease that affects fewer than 200,000 individuals in the United States” [2]. Men are more commonly affected compared to women, and the disease tends to occur more often in middle-aged patients but can affect patients of any age [3]. PF is also seen to occur more often in patients with epilepsy, diabetes mellitus, alcoholism with liver disease, repetitive trauma, and keloids [4].

PF is most often benign, but may cause pain and difficulty with ambulation and weight bearing as well as pain due to impingement of neurological or musculoskeletal structures as a result of the growing nodules [5]. The nodules are encapsulated, firm, and most commonly appear on the medial and central aspects of the plantar aponeurosis [6]. The formation of these fibromas is caused by the hyperproliferation of the fibrous tissue due to increased fibroblastic activity.

Treatment options

Symptom management is often the treatment of choice for plantar fibromas due to the characteristically benign nature. Some of these treatments include custom or over-the-counter orthotics to help with weight bearing, steroid injections, verapamil, radiation, extracorporeal shock wave therapy, tamoxifen hormonal therapy, and collagenase, all with varying degrees of success and scientific data to back up the effectiveness [1,7].

If symptom management does not alleviate the pain, or if the nodules continue to grow in size, surgical excision is the next option. There is a high rate of re-occurrence when only removing the fibroma itself, and for this reason excision of the entire plantar fascia is usually preferred [1,8]. Surgical management of this condition carries with it more significant specific complications including increased risk of hematoma, greater swelling, neurovascular damage and functional complications as well as potential painful scar formation negating the benefits of the surgery.

As with any surgical treatment, careful post-operative incision management is critical in preventing said complications at the incision site. There has been much research into the most beneficial post-operative wound management, particularly the most effective way to remove fluids such as blood and lymph from the area and keep the incision site clean. When fluids accumulate in the wound area, it can put pressure on the surrounding organs, nerves and vasculature, thus leading to pain and potential impairment of wound healing [9].

Surgical drains are used in many post-operative patients to remove excess fluids from wound sites through the process of the active suction of a drain placed during the procedure [9]. Another more recent incision management technique is negative pressure wound therapy (NPWT). This is a technique developed by Argenta and Morykwas in the early 1990s [10]. The aim of this therapy is to remove excess fluid and control the microenvironment of the wound through negative pressure suction by drawing the edges of the wound together.

One area of particular interest is that of a closed-incision negative pressure therapy (ciNPT) device such as Prevena™ by 3M + KCI (St. Paul, MN). This device acts on the same principles as NPWT, but rather on a closed incision with a constant -125 mm Hg pressure applied over the site continuously [11]. This is thought to help wound healing through an increase in blood flow, decreased stress on the incision, and removal of lymph from the local area. One meta-analysis of NPWT applied on closed wounds found this technique to be significant in reducing post-operative wound infections and seromas compared to standard post-operative dressings [12]. Stannard et al. in another study found that the use of ciNPT for post-operative management of high-risk lower extremity fractures resulted in a lower rate of wound dehiscence and infection [13]. While the results of these studies were favorable to ciNPT, there is not enough evidence in the current literature to recommend this technique for all wounds and all patients.

There is a vast array of available information on this therapy for surgeries performed throughout the body. However, the foot is one that is lacking representation in the literature, particularly the plantar surface, which poses unique surgical healing challenges. There are currently no universal treatment guidelines for the use of ciNPT devices such as Prevena™ in plantar fibroma excision surgery. The aim of this retrospective case series was to demonstrate and further define healing outcomes utilizing this specific ciNPWT device for this unique surgical problem.

## Case presentation

Case 1

**Figure 1:**
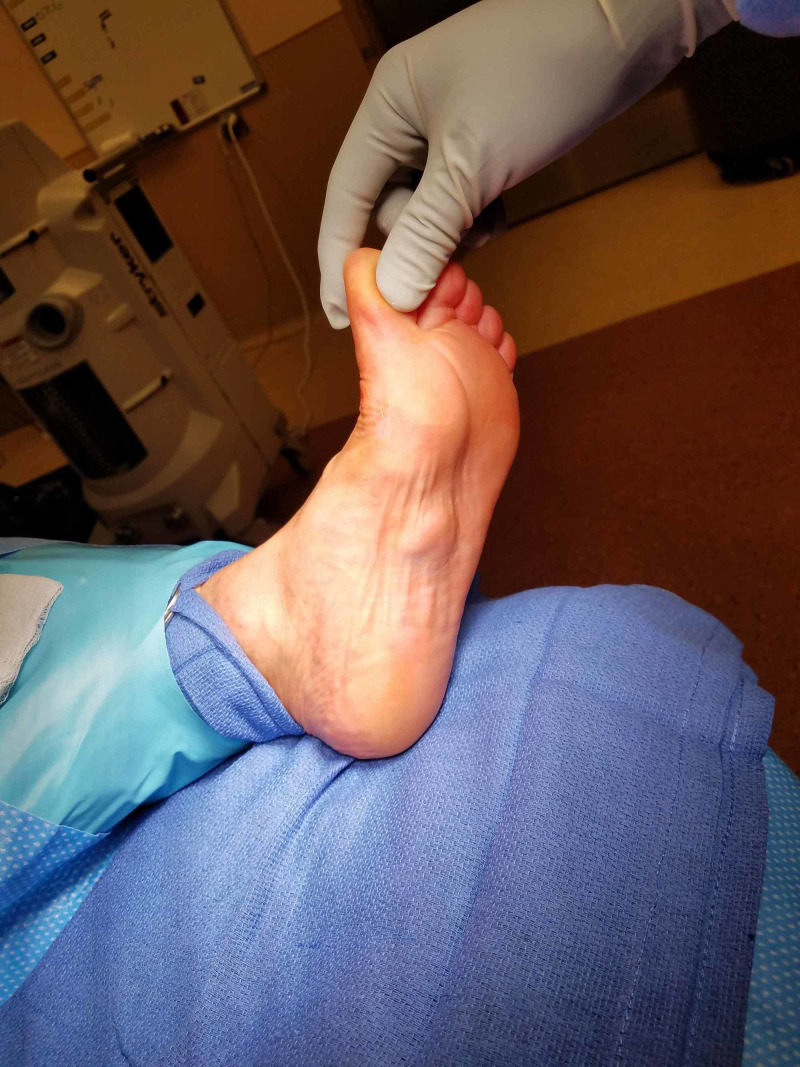
Case 1 pre-operative photograph.

**Figure 2:**
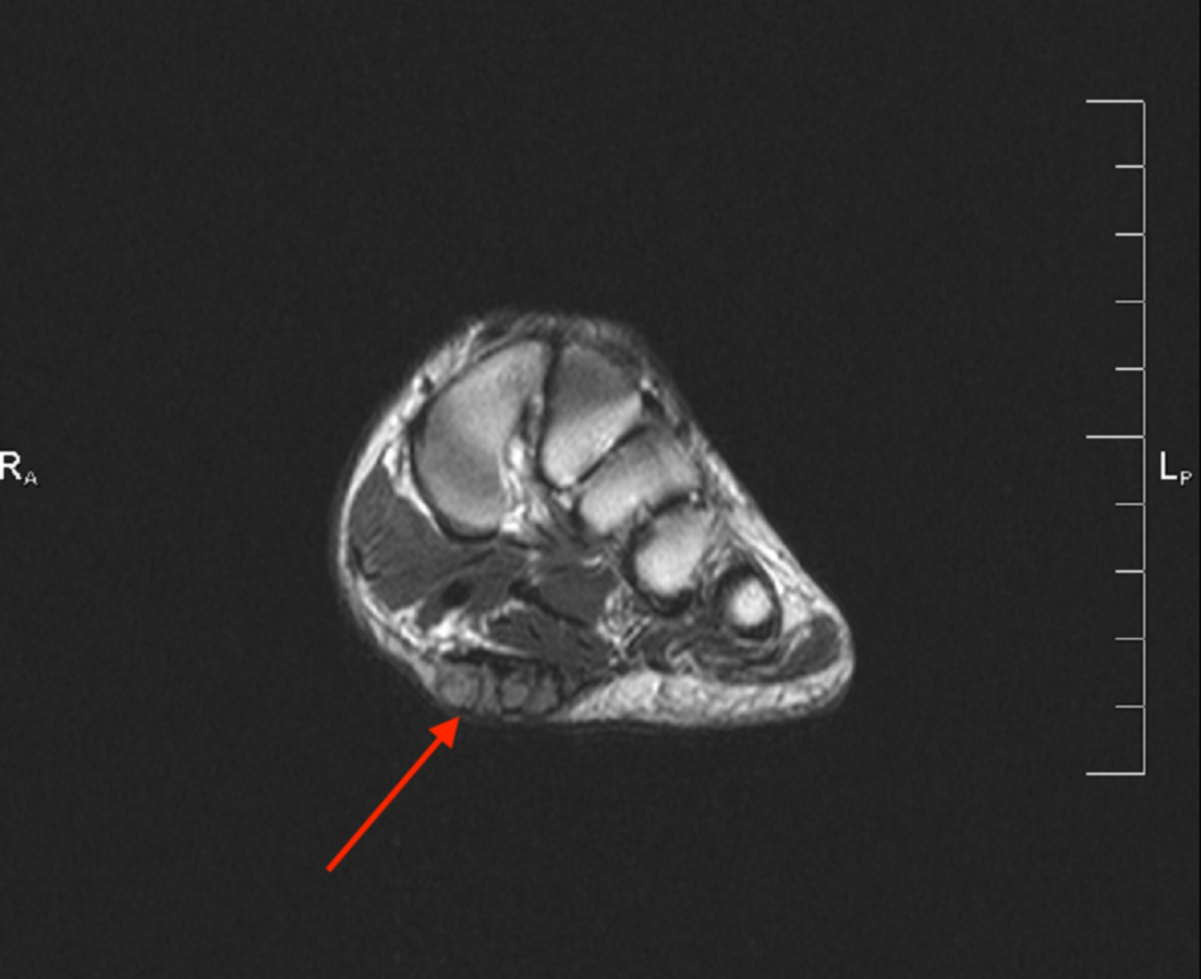
Case 1 MRI showing the axial T2 weighted image of the left foot demonstrating a plantar fibroma involving the central and medial cords of the plantar fascia beginning at the first tarsometatarsal joint.

**Figure 3:**
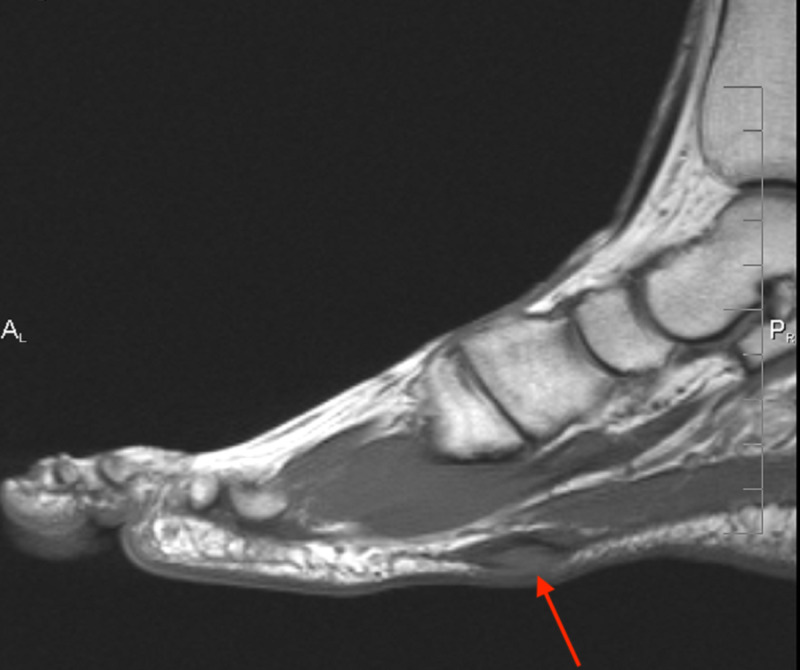
Case 1 MRI showing the sagittal T1 weighted image of the left foot demonstrating a plantar fibroma starting at the first tarsometatarsal joint with no involvement of the plantar musculature or flexor tendons.

**Figure 4:**
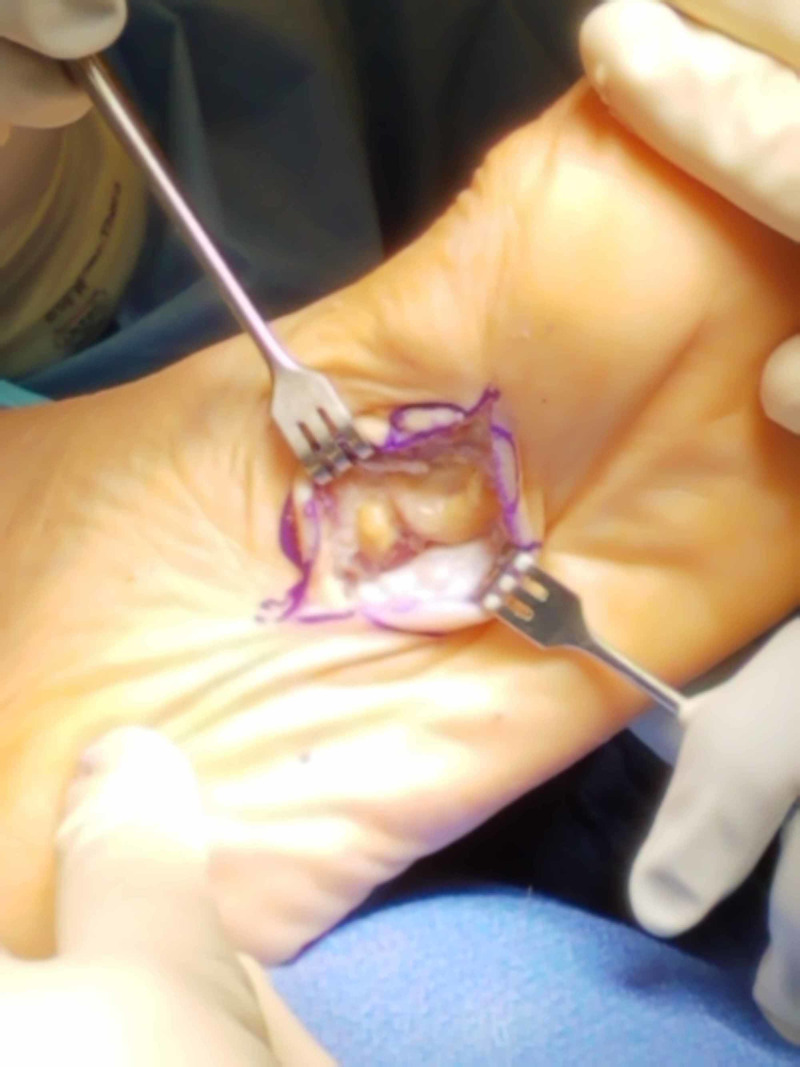
Case 1 intraoperative photograph.

**Figure 5:**
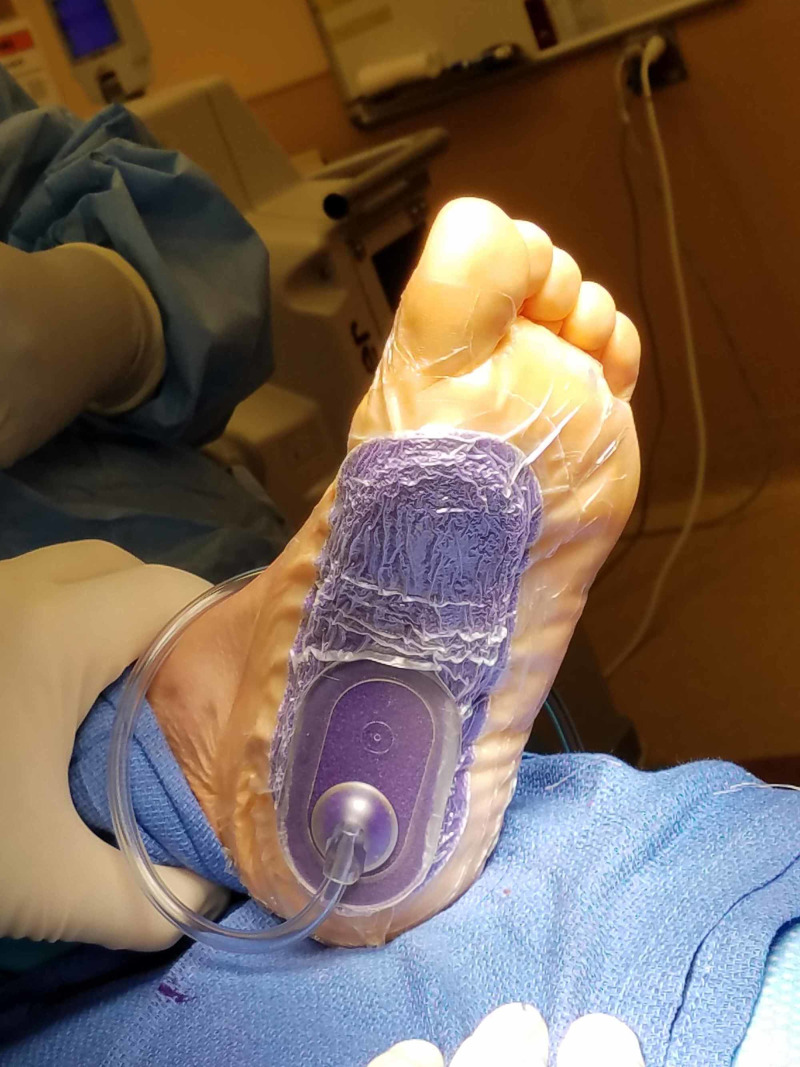
Case 1 demonstrating the intraoperative placement of the Prevena™ device.

A 56-year-old female presented for the evaluation of gradual onset bilateral soft tissue masses involving the plantar surface of both feet, with the left foot being significantly more symptomatic. Her past medical history included hypertension and high cholesterol. Social history included gainful employment in a moderately labor-intensive type job and several-year history of smoking. Her physical exam was essentially normal with the exception of somewhat firm, slightly mobile nodular masses involving both plantar foot surfaces in the region of the plantar medical arch (Figure 1). Tenderness was only noted involving the two masses on the left foot. Plain radiographs were negative. Conservative therapies were discussed and tried that included shoe gear and activity modification as well as analgesics. The patient declined injection therapy. An MRI was ordered that revealed two discrete soft tissue masses within the plantar fascia consistent with PF lesions without neurovascular encroachment (Figures 2, 3). Surgery was proposed and she wished to proceed with surgery on her symptomatic left foot (Figure 4). Risks and benefits were outlined for both complete fasciectomy and wide en bloc resection. The patient chose the latter technique and was advised to refrain from smoking that she did for several days both pre- and post-operatively. Surgery was completed without complications. Wound management included placement of a battery-powered ciNPT device (Prevena™) and light compression bandaging (Figure 5). The device remained in place for one week and then was removed. No significant erythema or swelling was seen. Surgical pathology was consistent with two, benign PF lesions. Sutures remained in place for a total of three weeks and were then removed. At this time, the incision was found to be negligibly tender, flat and well coapted. She was non-weight bearing immediately post-operatively through suture removal at which time she was transitioned to a walking boot with instructions to gradually increase weight-bearing activities to tolerance. Rechecks were unremarkable with the exception of a small area of dehiscence that was seen one week after suture removal. Complete incisional healing was appreciated about a week after suture removal. She returned to normal, daily activities including unrestricted work just over two months with no symptomatology or recurrence at the one-year mark.

 

Case 2

**Figure 6:**
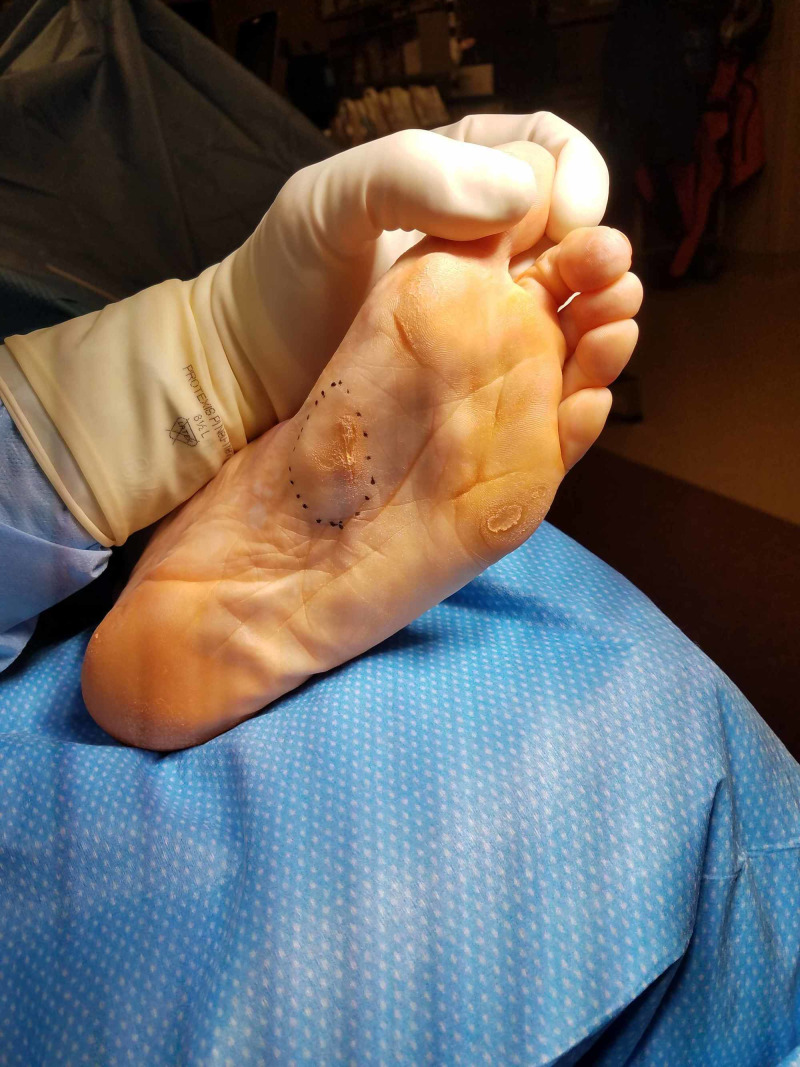
Case 2 pre-operative photograph demonstrating the recurrent plantar fibroma mass (note the old mildly hyperkeratotic scar).

**Figure 7:**
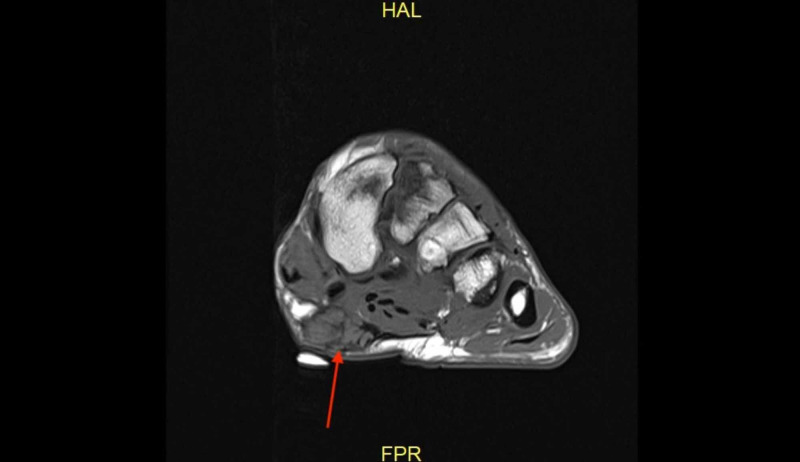
Case 2 MRI showing the axial T1 weighted image of the foot demonstrating a plantar fibroma of the midfoot, demarcated with a skin marker on the plantar aspect of the foot.

**Figure 8:**
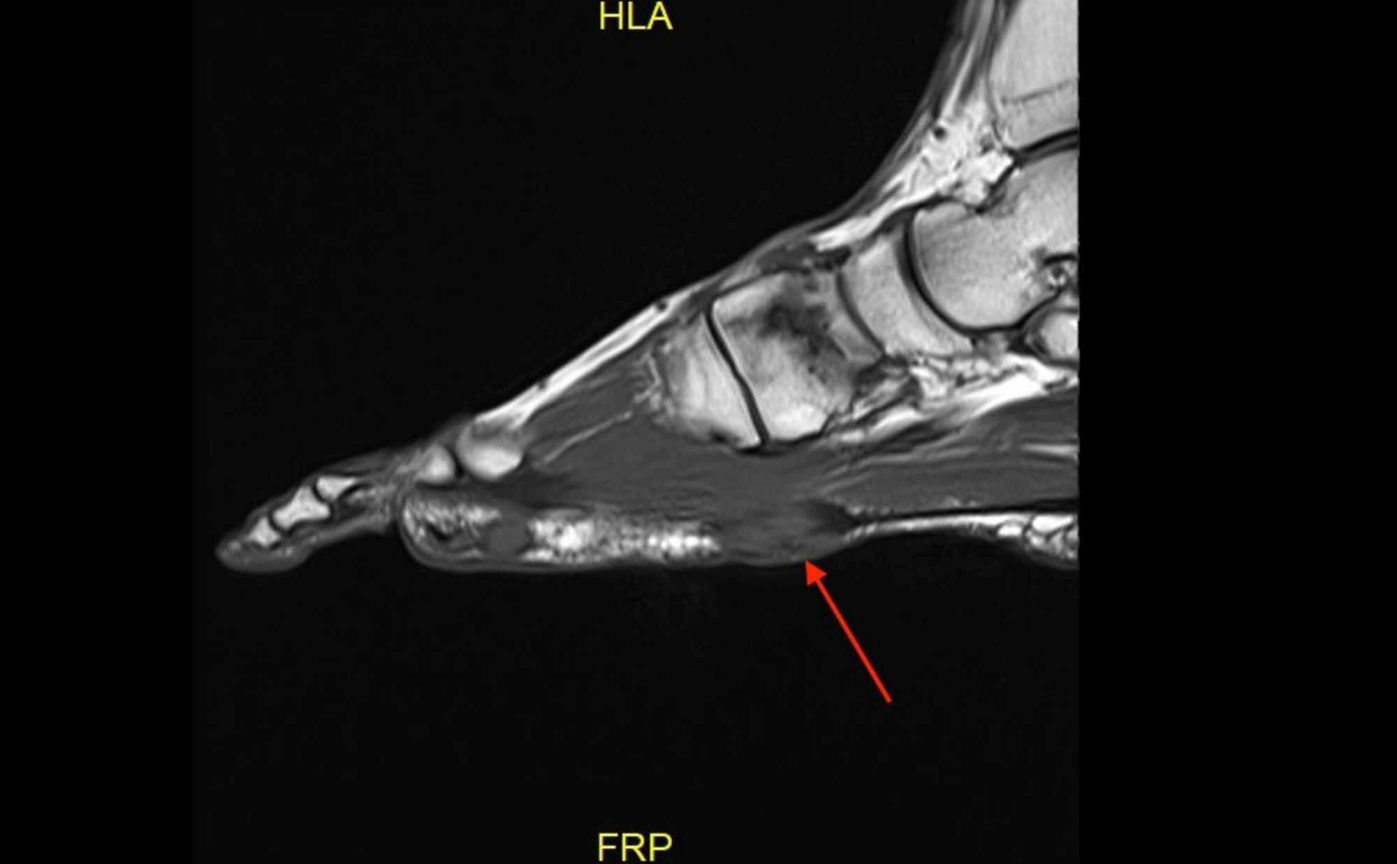
Case 2 MRI showing the sagittal T1 weighted image demonstrating a plantar fibroma of the first tarsometatarsal joint.

**Figure 9:**
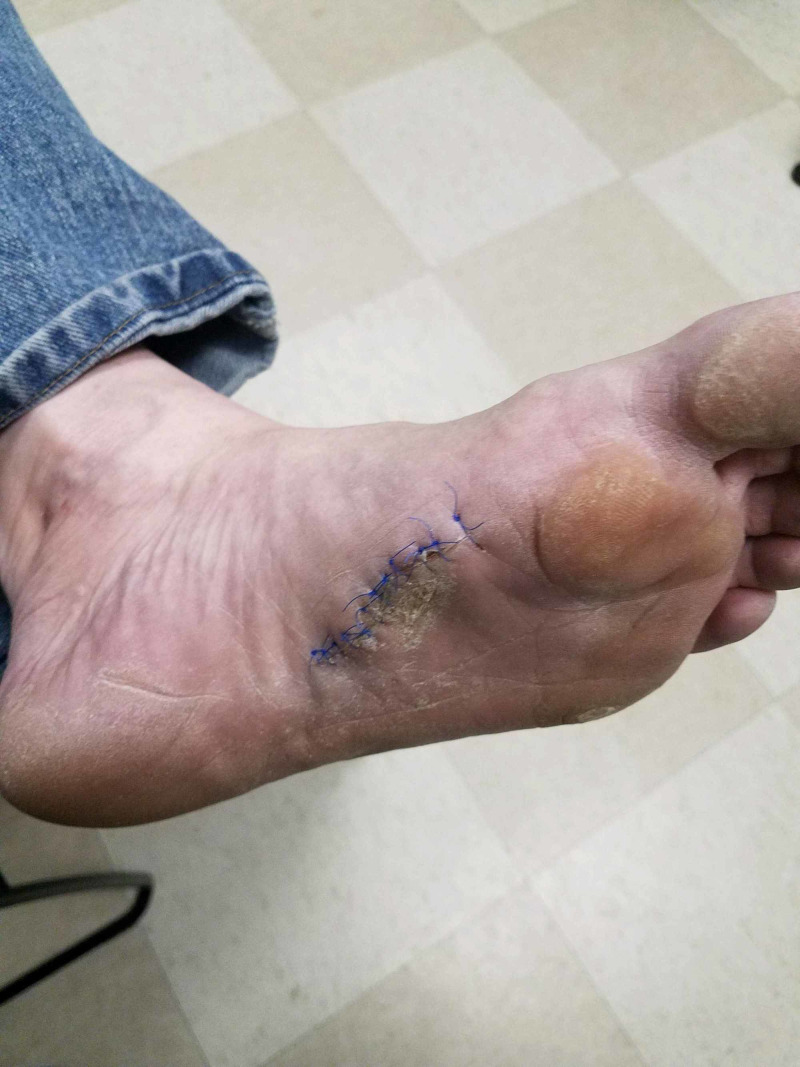
Case 2 one week post-operatively at Prevena™ takedown (note the lack of significant edema and the well-coapted incision).

Nearly a decade ago, a 58-year-old male presented for the evaluation of a painful soft tissue mass involving the left plantar foot. After conservative means were exhausted, he opted for surgery and elected to have a wide en bloc excisional removal of the mass. Pathology was consistent with PF. His post-operative period was uneventful and he remained asymptomatic for several years until November 2019. He presented again at this time for the evaluation of a gradually enlarging, symptomatic soft tissue mass in the same area as the original mass (Figure 6). Upon examination and further history, it was felt that this mass was consistent with a recurrent plantar fibroma. His past medical history was particularly remarkable for Type II diabetes and morbid obesity. Other problems included high cholesterol, anxiety, and depression. Social history included controlled substance abuse. His physical exam was normal. Specific exam of the soft tissue mass revealed a firm, tender, nodular mass involving the left plantar medial arch in the same region of a well-healed, mildly hyperkeratotic surgical scar. Plain radiographs were negative. When he opted for surgery, the risks and benefits were outlined of both complete fasciectomy and wide en bloc resection. He chose the latter technique. An MRI was ordered that revealed changes consistent with a PF mass (Figures 7, 8). Surgery was completed without complications. Wound management included placement of a battery-powered ciNPT device (Prevena™) and light compression bandaging. The device remained in place for one week and was then removed. No significant swelling or erythema was seen (Figure 9). Surgical pathology was consistent with a solitary benign plantar fibromatosis lesion. Sutures remained in place for a total of three weeks. At this time, the incision was found to be negligibly tender, flat and well coapted. Partial weight bearing in a walking boot was initiated. At two weeks from the time of suture removal, he was transitioned to full weight bearing. At just over three months, he was discharged from care symptom free without signs of recurrence.

 

Case 3

**Figure 10:**
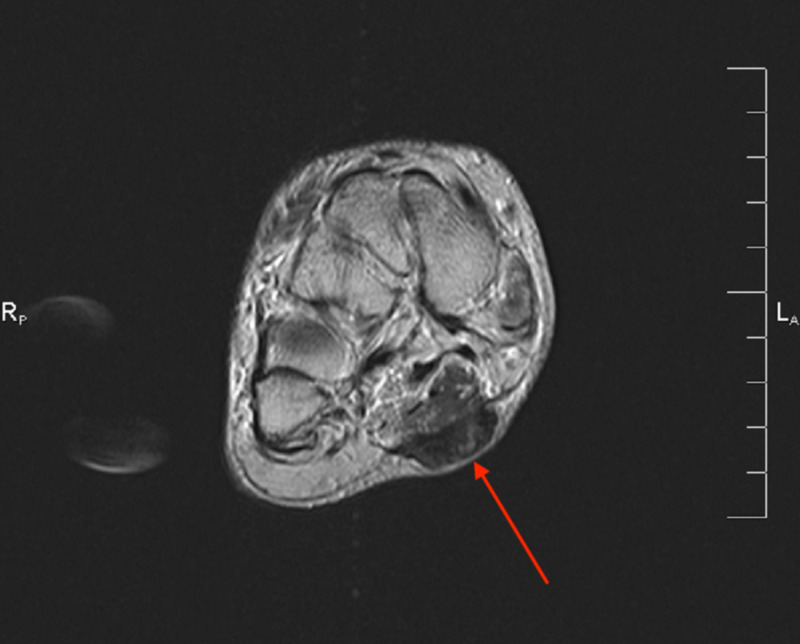
Case 3 MRI showing the axial T2 weighted image of the right foot demonstrating a large plantar fibroma extending from the central cord into the subcutaneous tissue of the plantar surface of the foot.

**Figure 11:**
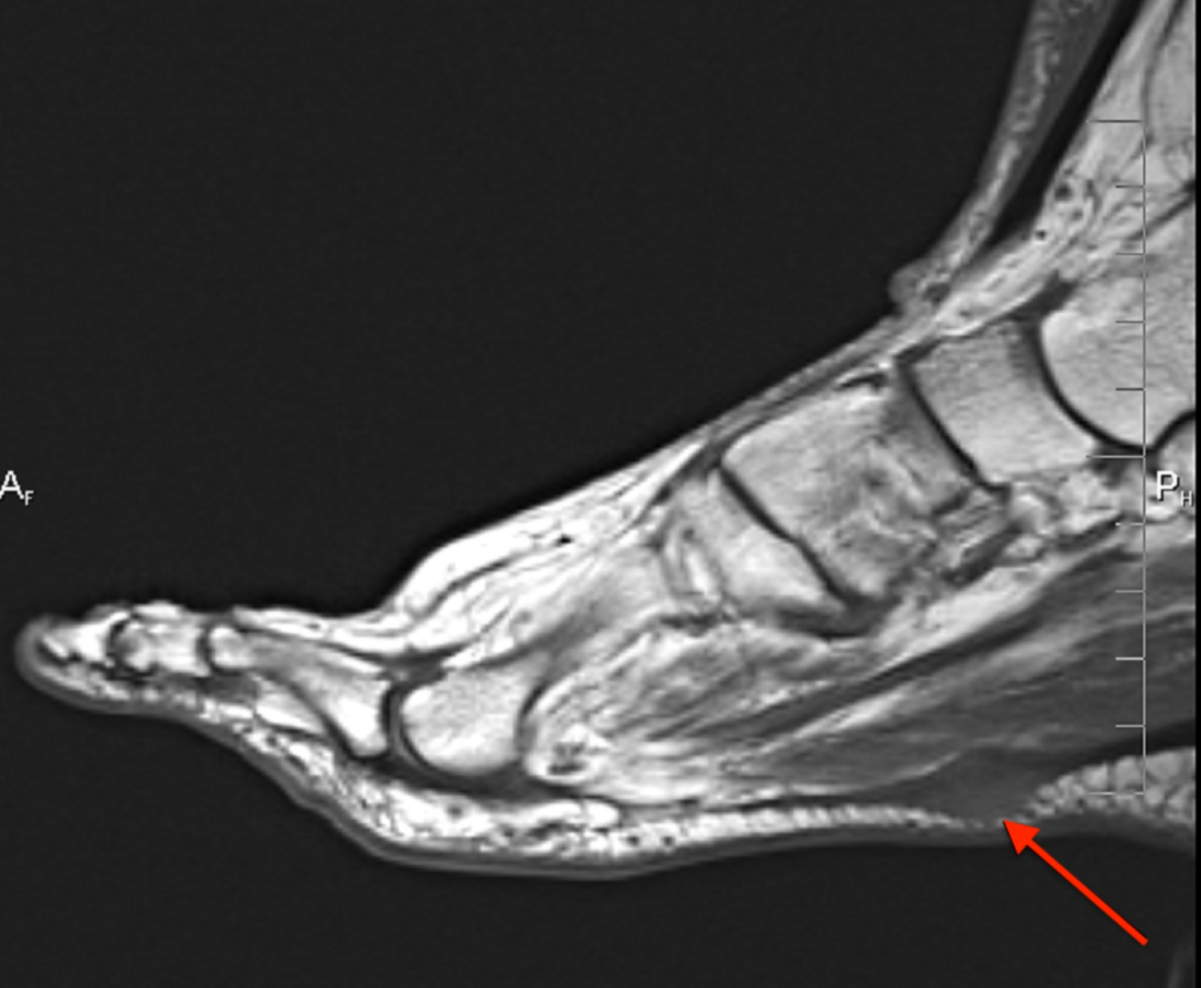
Case 3 MRI showing the T1 weighted sagittal image of the right foot demonstrating a plantar fibroma within the central portion of the plantar cord.

**Figure 12:**
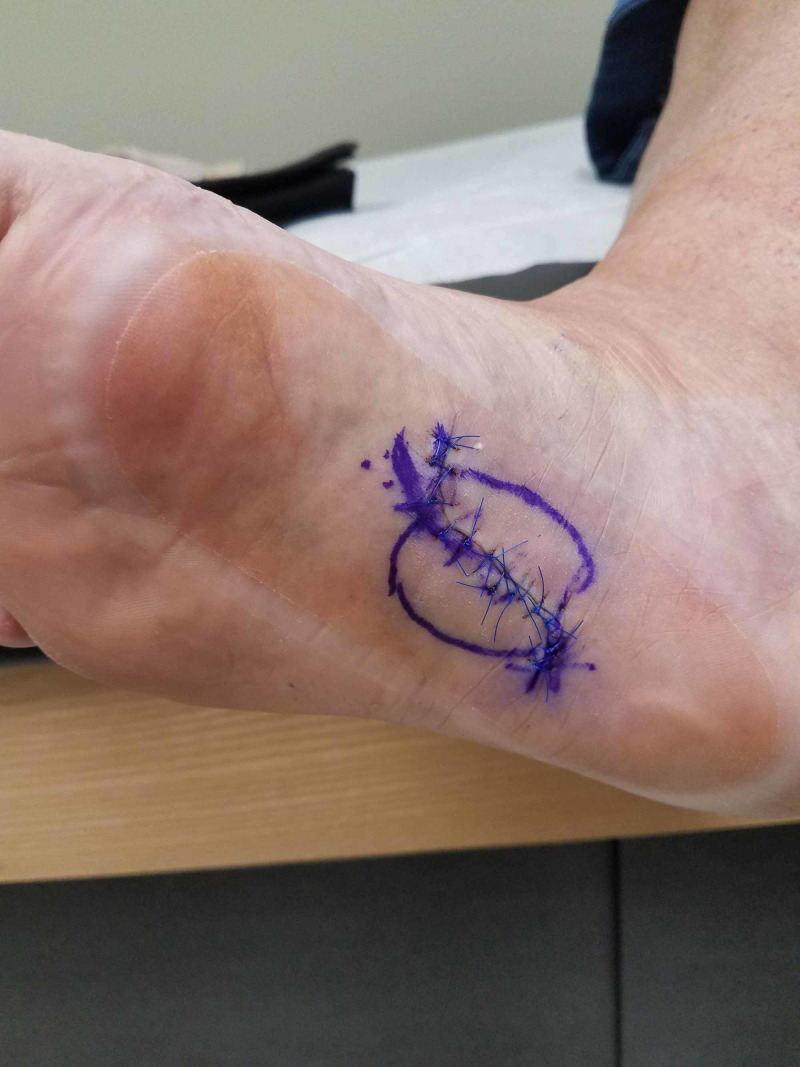
Case 3 one week post-operatively at Prevena™ takedown (note the lack of significant edema and well-coapted incision).

**Figure 13:**
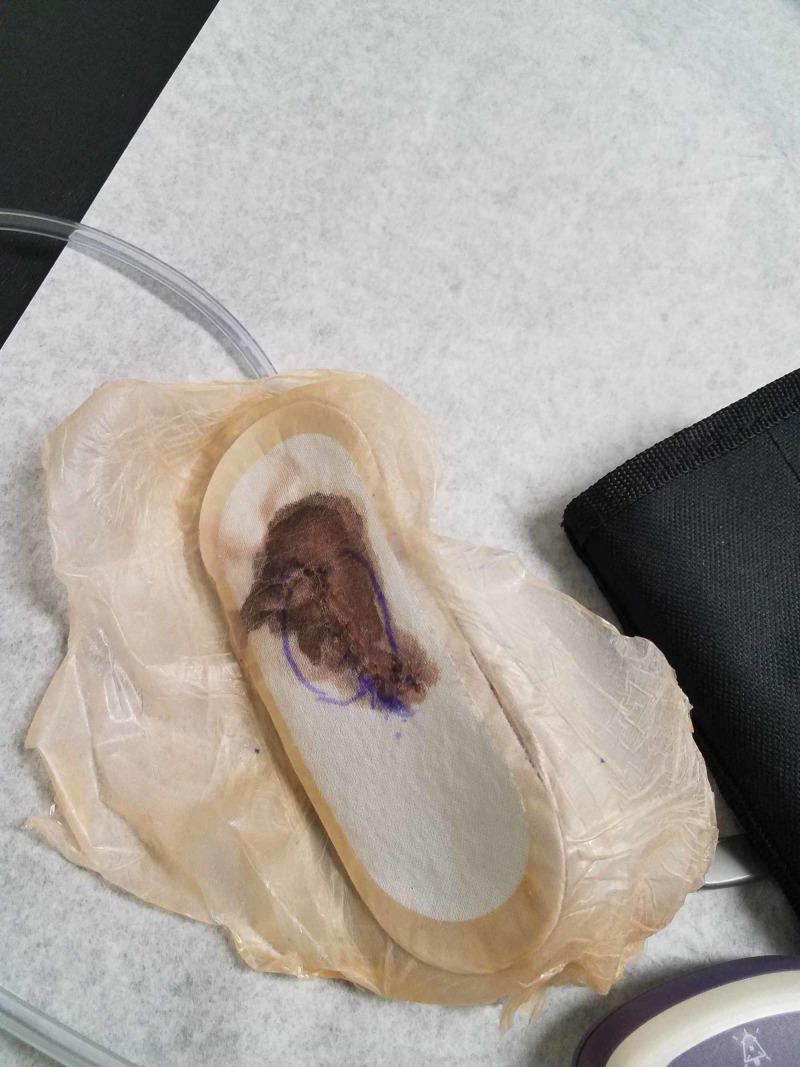
Case 3 one week post-operatively showing Prevena™ dressing (note serosanguinous dressing capture without extravasation or strikethrough).

A 62-year-old female presented for the evaluation of a symptomatic soft tissue mass involving the right plantar medial arch. Her symptomatology was relatively insidious by history and what prompted her visit was gradual intolerance of a certain shoe gear. She reported the mass was present for over a year but symptomology escalated about four months prior to initial evaluation. Her past medical history included hypertension, thyroid disease, Factor V Leiden pathology with a history of deep vein thrombosis, degenerative joint disease, and morbid obesity. Social history was unremarkable. Her physical exam was largely unremarkable except for significant pes planovalgus changes in both feet and a painful mass involving the right foot. Specific examination of the soft tissue mass revealed a mildly firm, slightly mobile, tender soft tissue mass in the medial plantar arch. Plain films demonstrated no acute process. Both non-operative and operative treatment options were offered and the patient chose the latter, electing for wide en bloc excision instead of complete fasciectomy. Prior to surgery, an MRI was ordered that demonstrated changes consistent with a solitary plantar fibroma lesion (Figures 10, 11). Her surgery was without compromise and included perioperative anticoagulation bridge management in light of her history of deep vein thrombosis. Wound management included placement of a battery-powered ciNPT device (Prevena™) and light compression bandaging. At bandage takedown, the incison was observed to be well coapted with no complications (Figure 12). The device remained in place for one week and was then removed. No drainage strikethrough was seen (Figure 13). Surgical pathology was consistent with a solitary benign plantar fibromatosis lesion. Sutures remained in place for a total of three weeks. A walking boot was used for two weeks after suture removal. Healing progressed uneventfully with gradual increase in weight bearing and activities. Dorsal foot swelling on the operative foot persisted but eventually resolved. She ultimately returned to normal shoe gear at three months and was discharged from care without symptoms or signs of recurrence at six months.

 

## Discussion

Although the National Institutes of Health classify PF as a “rare” condition, this condition is certainly encountered in private practice [2]. Developments in the non-operative intervention are ongoing with various degrees of success and shortcomings demonstrated in the literature. It is well accepted that conservative, palliative, and interventional non-operative measures should be considered as first-line treatments reserving surgery for more symptomatic cases. As stated previously, plantar fibroma surgical complications, with wound healing in particular, are not uncommon and are related to the depth and breadth of the operation. One retrospective study found that 52% of post-operative plantar fibromatosis sites experienced delayed wound healing with a potential need for a skin graft [14]. Post-operative measures to protect the incision and minimize swelling are paramount to lessen the chance of wound-healing surgical complications. Techniques such as compression bandaging, non-weight bearing during incisional healing, and drain placement have been utilized successfully to decrease swelling and remove fluid at the surgical site. We have presented three cases in which a battery operated closed-incision negative pressure device (Prevena™) was used with favorable outcomes in lieu of surgical drain placement. This device is designed to manage the closed surgical environment by drawing fluid away from the operative site, therefore lessening the chance of serosanguinous fluid collection beneath the incision. Such fluid collections can potentiate problems that include tension at the incision site resulting in dehiscence or pressure necrosis. Fluid collections, if unchecked, can also serve as a nidus for infection. In this regard, Prevena™ aids in reducing the incidence of surgical site infection by decreasing the chance of fluid collections in patients at high risk for post-operative infections [11]. All the three patients in this case series had co-morbidities that put them at higher risk for plantar fibroma surgical complications. These included smoking, diabetes, obesity, and Factor V Leiden. All three patients demonstrated favorable outcomes without significant wound-healing complications. The one exception was a subtle dehiscence in the patient with the smoking history; however, the dehiscence healed uneventfully without complications.

## Conclusions

Several treatment options are available for PF. These include palliative measures, non-operative interventions, and surgery, all with varying degrees of success and complication risk. We presented three cases of surgical wide en bloc resection of PF lesions in which a battery-powered ciNPT device (Prevena™) was used successfully in lieu of surgical drains. All three patients demonstrated favorable outcomes without significant complications. While additional studies are necessary, we believe this plantar fibroma surgical case series demonstrated that the use of a battery-powered ciNPT device (Prevena™) was beneficial in reducing wound-healing complications such as swelling, fluid collections beneath the surgical site, and surgical site infection.
